# Multi-species collapses at the warm edge of a warming sea

**DOI:** 10.1038/srep36897

**Published:** 2016-11-17

**Authors:** Gil Rilov

**Affiliations:** 1 National Institute of Oceanography, Israel Oceanographic and Limnological Research, PO Box 8030, Haifa, 31080, Israel

## Abstract

Even during the current biodiversity crisis, reports on population collapses of highly abundant, non-harvested marine species were rare until very recently. This is starting to change, especially at the warm edge of species’ distributions where populations are more vulnerable to stress. The Levant basin is the southeastern edge of distribution of most Mediterranean species. Coastal water conditions are naturally extreme, and are fast warming, making it a potential hotspot for species collapses. Using multiple data sources, I found strong evidence for major, sustained, population collapses of two urchins, one large predatory gastropod and a reef-building gastropod. Furthermore, of 59 molluscan species once-described in the taxonomic literature as common on Levant reefs, 38 were not found in the present-day surveys, and there was a total domination of non-indigenous species in molluscan assemblages. Temperature trends indicate an exceptional warming of the coastal waters in the past three decades. Though speculative at this stage, the fast rise in SST may have helped pushing these invertebrates beyond their physiological tolerance limits leading to population collapses and possible extirpations. If so, these collapses may indicate the initiation of a multi-species range contraction at the Mediterranean southeastern edge that may spread westward with additional warming.

In this Anthropocene, species are being lost from Earth at an alarming rate^1^, but extinctions or even extirpations (local or regional extinctions) are usually restricted to relatively small or isolated, often endemic, populations, or to high trophic level, slow-growing species with low reproductive rates^2^. In the marine environment, sharp population declines of highly abundant species were typically associated with extensive habitat destruction or overharvesting (e.g., overfishing of large pelagic predators, refs 3,4). Recently, population collapses are also reported in relation with extreme climate events^5^, normally, at the warm edge of species distributions; for example, dramatic community changes at the northern edge of the West Antarctic Peninsula^6^, or the collapse of kelp beds and their associated communities in Western Australia due to a short, acute, increase in temperatures^7^,^8^,^9^. But such reports are still rare.

The southeastern Mediterranean (Levant) coast (Fig. 1) is potentially a region where extensive, non-harvesting-related decline of coastal species may occur. This is because it is the southeastern (trailing) edge of distribution of most Mediterranean and Atlanto-Mediterranean species. The coastal water conditions are naturally extreme, being the hottest, saltiest, and having the lowest primary productivity in the Mediterranean^10^. Due to its relatively small size, the eastern Mediterranean basin also responds promptly to atmospheric variability^11^. Paleo-oceanographic reconstruction of sea surface temperature (SST) on the Israeli coast produced a continuous high-resolution millennial-long record that showed a warming trend between 1750–2010 of about 2 °C (that have accelerated in the last 3 decades), which mainly reflects Northern Hemisphere changes in air temperature that are amplified for this specific region^12^. Satellite data analysis suggested a rapid increase of almost 1 °C in the eastern Mediterranean SST between 1985–2006^13^. More recent analysis of temperatures measured *in-situ* >50 kms offshore the Israeli coast, at the southeastern corner of the Levant basin, suggested an even greater SST warming of about 3 °C between 1980–2013^14^. In such a naturally-extreme environment that is becoming more extreme, native species may be more vulnerable to stress – either regional (e.g., ocean warming) or local (e.g., pollution, diseases) – that can trigger population collapses and possibly extirpations. Because the Mediterranean Sea can be regarded as a miniature ocean^15^, many of the processes occurring in it over the past few decades can also provide insights on the resilience of marine ecosystems worldwide^16^. So far, Non-harvesting related population collapses were reported almost exclusively from the northwestern Mediterranean and were linked mainly to strong heatwaves in the past two decades^17^,^18^. The Levant, at the southeastern edge of the sea, is known as a prime hotspot for species invasions since the opening of the Suez Canal in 1869 (hence from the warmer Red Sea/Indian Ocean), and much has been reported on species “additions” there^19^,^20^,^21^, but relatively little is known on recent native (non-harvest related) species collapses or extirpations in the region (although some population declines were suggested to be related to exclusion by invaders ref. 19). Therefore, evidence of population collapses from this region is of importance for the understanding of biological shifts in this age of rapid environmental and ecological change.

Unfortunately, decadal time-series for any southeastern Levant coastal species do not exist to enable an examination of continuous temporal population trends. But, reconstruction of substantial demographic changes, at least for some taxa, can potentially be done if reliable data on present-day species status is available and different sources of information on their past status exist, to allow comparison (for example, ref. 22). Such sources can include sporadic surveys that consist of one or a few data points (see for example refs 23,24), taxonomic information with some reference to relative abundance, even if descriptive, and museum records^8^,^25^, as well as local knowledge that can be assessed with questionnaires targeting naturalists and professionals (fisherman, divers and academics) alike^26^,^27^,^28^. Using this approach, in this study, I compare data from recent (2009–2015) comprehensive rocky intertidal and subtidal biodiversity surveys (thousands of quadrats and hundreds of dives) on the Israeli coast (Fig. 1), as well as three “reference” sites further west surveyed in 2013 and 2014, to (1) data from past sporadic surveys (conducted in the 1970 s and 1990 s) of four once highly-abundant, ecologically-important invertebrate species that are not commercially or artisanally harvested, (2) abundance categories in a detailed Levantine mollusca taxonomic publication based on past semi-quantitative surveys conducted between the 1967–77^29^,^30^, (3) malacological museum records, (4) survey of local knowledge on one taxon (urchins) conducted in 2015. I also compiled remote-sensing and nearshore *in-situ* temperature data to verify the recent warming trends of the southeastern Levant coastal waters.

## Results

### Population collapses

Comparing past and present data of four previously highly-abundant native species for which some survey data exists indicates their total population collapse (to zero or near zero abundance) in the past 2–3 decades. In the large whelk *Stramonita haemastoma*, significant signs of rapid decline were already evident in the late 1990’s when populations were still abundant but no recruitment of young was seen for five years^31^, leading to a total collapse, demonstrated here by its complete absence in the last seven years (2009–2015) in Achziv, where it was most abundant in the 1990 s (Fig. 2a). In all intertidal and shallow subtidal transect surveys conducted along the entire coast over the past seven years (382 and 270 transects, respectively) not a single live individual was recorded, and only three very large (8–10 cm) and seemingly old (shell highly eroded) individuals were found in a shallow artificial lagoon at Akko in 2010, outside the transects. Since then, this relic population has also gone extinct. The whelk was not recorded in both the Cyprus and Crete surveys, but was relatively abundant in two sites near Naples in southwestern Italy where it was highly aggregated (average of 1.6 individuals per m^2^ at 5 m depth).

The once highly-abundant purple sea urchin, *Paracentrotus lividus*, as well as its less abundant relative, *Arbacia lixula*, are all but extinct now along the coast, as exemplified in the *P. lividus* densities measured inside the Achziv marine reserve (Fig. 2b). In Dothan’s^32^ 1970 s surveys, *P. lividus* densities on horizontal surfaces on the west side of the Achziv Islands were around 12 individuals per m^2^. At the exact same area, close to 80 1 × 30 m transects were conducted between 2010–2015, and only one urchin was encountered. A thorough circling of the tiny rocky islets in May 2011 in search for urchins revealed 9 more individuals, and in all other dives, ten more individuals were observed in shallow waters near Haifa, outside transects. In shallow (<5 m) surveys conducted in 1994 at several rocky sites along the central and northern coast, *P. lividus* densities ranged between 0.5–1.9 per m^2^ (Fig. 2b), indicating that it was still relatively abundant in the mid-1990s. In the 2013–14 Cyprus, Crete and southern Italy surveys, *P. lividus* density ranged between 0–2.1 per m^2^. In Dothan’s surveys in Achziv in the mid-1970s, the black urchin, *A. lixula*, was reported to have an average density of 0.46 per m^2^, but in the hundreds of dives conducted in the present-day surveys, only one individual was seen (in August 2014 near Haifa). This species was not observed in the more limited Cyprus and Crete surveys.

The reef-building, sedentary, snail, *Dendropoma petraeum*, was abundant at many vermetid reef sites along the coast in the 1990 s, though some rim degradation was noticed and a possible decline in its populations was suggested^33^. The species was still present in the early 2000’s^34^, but in the surveys of the past seven years it was extremely rare along the entire coast, as was also noticed in 2012 by Galil^35^ in two sites. Its collapse is exemplified in the Habonim site where more than 40% of the rim (the elevated reef edge created by the vermetid) length was covered by live *D. petraeum* in the mid 1990 s, but between 2009–15 its live cover there was near zero (Fig. 2c). Between 2013–15, a few individuals and small clusters (<100 cm^2^) were seen in Achziv and Habonim but they did not sum up to more than 0.05% of the length of the rim. In contrast, in Halavro cave, northwest Cyprus, *D. petraeum* aggregations occupied about ~50% of the length of the reef edge, and there were small live patches (0.6%) in the southeast site, Liopetri. In one north (Alykes) and one northwest (Falasarna) site in Crete, *D. petraeum* created extensive but thin patches (there are no rims in these reefs) with a cover of 60% and, 12.2%, respectively. No vermetid reefs were found in Naples and Ischia (beyond the northern limit of vermetid reefs ref. 36), but a few small live aggregations were seen in Ischia.

On subtidal rocky reefs, a mere 0.1 and 1.6% of all counted macro-epi-benthic (living on the surface of the rocks) bivalves and shelled gastropods, respectively, were of native species, and the overwhelming majority was of invaders of Indo-Pacific origin (Fig. 3). Based on the detailed description in the taxonomic literature^29^, and on malacological museum records, it can be safely assumed that Israeli Mediterranean reefs used to be occupied by many abundant native bivalves and gastropods that are now absent or extremely rare. Of the 18 native bivalves, 38 native gastropods and three native polyplacophora species described in the Levantine malacological literature as common or very common on reefs^29^, 11 (61%), 24 (63%) and 2 (66%), respectively, were not found at all in the present-day surveys (Fig. 4, see details in Supplementary Table S1). The bivalves *Chama gryphoides, Anomia ephippium* and *Petricola lithophaga* were not recorded in our surveys, however they were brought live to the Tel Aviv University malacological collection after 2010, (Table S1) meaning that they were still present during our recent sampling period. The first two bivalves are relatively small and cemented to the rock and can also be heavily fouled and the third lives mainly within rocks or crevices; thus they may have been missed during the visual surveys. It should be noted that the native gastropod *Columbella rustica* (1.2% of the total count, Fig. 4) was under-represented in our underwater surveys because it is found almost exclusively within macrophyte meadows (and thus hard to enumerate non-destructively). Such meadows are, today, very restricted on the reefs but macrophyte samples taken to the lab indicated that this species is still quite abundant within the meadows (Rilov, unpublished data). In the “live-rock” sampling (see Methods) the native rock-boring species, *Lithophaga lithophaga* and *Striarca lactea*, were the only bivalves occupying cavities within rocks, and they occurred in 63–79% of the samples. This means that the single shallow rocky habitat where native bivalves dominate today is inside biogenic rocks comprising the reefs.

In the local knowledge survey on urchin population status and trends, ninety nine people responded within a week. Of them, 63 declared familiarity of >10 years with the Mediterranean Sea and only their answers were analyzed. Academics in marine science represented 18% of the responders, 44% were recreational divers, 10% were professional divers, and the rest were fishermen (professional or recreational) and Others. Regarding the status of the urchins, 57% responded that there are no urchins today, 30% reported that there are much less than before, 5% reported no change and 8% reported more urchins than before. The main timing of change in urchin populations that responders (only those reporting decrease) indicated varied from before the 70’s (8%), the 80 s (22%), the 90 s (33%) and the 2000 s (37%), suggesting that in most responders’ view it was rather recent. Of those with experience of over 30 years (23) 91% thought that the decline started from the 1980 s or later and 53% of those thought it started from the 1990 s onward.

It should be noted, that collapses of non-harvested species in the southeastern Levant may not be restricted to invertebrates, which are the focus of this paper. For example, the intertidal macroalga, *Halimeda tuna*, was present in Mikhmoret (central coast) in the 1960 s^37^, was still recorded there as well as in many other sites (~10% frequency of occurrence in 1262 sampled quadrats) in macroalgal surveys conducted at 19 locations between 1973–1995^38^, but was absent in a 2000 study in Mikhmoret^39^ as well as in the present monitoring surveys (all 11 sites, all six years, 3120 intertidal reef quadrats). It was also not found during the Cyprus and Crete expeditions, but these surveys were of more limited spatiotemporal scope, so this finding should be taken with caution.

### Ocean warming

Satellite data analysis of decadal changes for 1980–2010 along Israeli coast revealed a 0.27–0.47 °C warming per decade (Fig. 5a), matching Levantine rates from the earlier, shorter, satellite time series^13^. Coastal warming rates were highest during early summer (for southern coastal sections) and early winter (November-December) at most coastal sections (between 0.4–0.47 °C per decade). Summer (August) temperature fluctuate considerably until 1998, after which values are maintained above 28 °C (Fig. 5b). Furthermore, the number of days/year warmer than 95% of SSTs has increased by 14 days per decade, indicating increasingly longer peak summer conditions, as evident in many other coastal areas worldwide^40^. Temperature loggers on buoys 2-km offshore also indicate an increasing SST trend in the peak of summer (Fig. 5b). Again, it seems that the major shift in temperature occurred around 1998 (after which mostly positive deviations from the long-term mean occur, Fig. 5c). The average August temperature for 1998–2015 was 1.2 °C higher than that for 1992–1997 (29.3 to 30.5 °C; two-tail t-test p = 0.0001, in the southern site, Ashdod). Furthermore, maximum temperatures in the southern location exceeded 32 °C in four years between 1998–2015 and approached or crossed 31 °C six times in the northern site, Haifa, during that period (Fig. 5b). Although the temperature recorded by remote-sensing for the north and south coastal pixels (where the buoys are located) strongly correlate with that measured by the nearshore buoys (r^2^ = 0.58 and 0.80, respectively), the values of the latter were higher by almost 2 °C for the same period (Fig. 5d), indicating that remote-sensing data is considerably underestimating the temperatures that coastal species actually experience in this region. The sharp shift in average summer temperature in 1998, seen in both the satellite and buoy data, may be related to the dramatic and sudden change in sea surface structure (presumably associated with the relaxation of the Eastern Mediterranean Transient event) that occurred in 1997^41^. The last two incidences of near 32 °C (the peak of summer of 2010 and 2012) seen in the buoy data, were also recorded at 0.5 m depth in the shorter time-series at the four rocky shore monitoring sites (Fig. 6). By comparison, shoreline summer maximum temperature (mid-August) measured in 1976 at a similar depth at the north site of Achziv was only 29.0 °C^32^.

## Discussion

Given the evidence shown in this study for major population collapses (and plausible extirpations) of dozens of invertebrates and one algae, it is highly probable that many other taxa from even less-studied groups have seriously declined in the southern Levant—unnoticed. But what are the causes for these multispecies collapses? At this point, it is impossible to directly relate most of the extensive collapses documented here to climate change. I therefore first discuss the four other major known drivers of biodiversity loss—habitat destruction, pollution, over-harvesting and bioinvasions^42^—to examine their relevance to the specific study region (the Israeli Mediterranean coast), the specific study habitat (coastal reefs) and the specific taxonomic groups examined in this study (reef sea urchins and molluscs). Habitat destruction is not relevant here because reefs have not been considerably structurally-compromised along the Israeli coast except in some vermetid reefs where the rim seems eroded due to the disappearance of *D. petraeum*^33^ (personal observations). With regard to pollution, contaminants mostly occur in the Haifa Bay and levels in most categories (e.g., heavy metals) have in-fact been declining or stabilizing in the past two decades^43^. Eutrophication is also generally not a major stressor along most of the open Israeli coast. This region is considered ultra-oligotrophic^44^, and water samples taken monthly at the four core monitoring sites (see Methods) for the past six years indicate that nutrient and chlorophyll levels are indeed very low with no significant sign of eutrophication (e.g., chlorophyll *a* levels varies between 0.1–0.9 μg/l, Rilov unpublished data). Over-harvesting is not directly relevant either because, at least in the past century, none of the collapsed species have been commercially harvested in Israel. One cannot exclude the possibility of indirect causes of overharvesting of commercial or bicatch species through trophic-cascades whereby overharvested predators or herbivores affected the food-web dynamics or even the habitat, resulting in strong negative effects on the associated species, among them, the species discussed here. The scale and rate of bioinvasions are indeed extensive in the eastern Mediterranean^19^, especially along the Levantine southeastern coast^45^, and they have been claimed to have major negative ecological impacts on the regional biota^45^; a claim that makes much sense but is speculative without experimental evidence. However, bioinvasions are not directly relevant to the collapse of *D. petraeum* and *S. haemastoma* that do not have any obvious non-indigenous competitors or predators. In fact, an intertidal mussel invader, that is favored by *S. haemastoma*^46^ over native prey, has dramatically increased in abundance since the 1990 s^33^, which should have theoretically boosted its populations. Competitive exclusion by alien herbivorous fish may have contributed to the urchin collapse. Two herbivorous Red Sea siganid fish, *Siganus rivulatus* and *S. luridus*, are abundant on Levant rocky reefs^47^, and were shown in Turkey^48^ and suggested in Greece^49^ to create subtidal ‘barrens’ in urchin-less rocky areas by removing all erect fleshy algae. Barrens today are the dominate feature on the Israeli reefs as documented in most surveys (Rilov, unpublished data), and the fish effects on the macroalgae were experimentally shown also in Israel^50^. It is possible that the fish remove favorable algae to the degree that affects the urchins’ fitness, slowly reducing their population viability in the region. However, in the central Aegean Sea, where water is cooler than along the Israeli coast, *S. luridus* and *P. lividus* still co-occur in high numbers even though turf-barrens are present^49^, reinforcing the suggestion that competition with the fish is not the main cause of the urchin collapse. The current rarity of urchins in the southeastern Levant seems contrary to trends in many parts of the western Mediterranean, where urchins increased in numbers due to overfishing of their main predators (resulting with barrens due to overgrazing), although increase in temperature was also speculated to increase their abundance and activity^51^,^52^. In other Mediterranean areas, extensive harvesting of urchins for consumption was shown to reduce their populations^53^; but there is no harvesting of urchins in Israel that could explain their decline. Of course, it is quite plausible that of the many mollusc species that have collapsed on the reefs, some may have been out-competed by invader counterparts, as has been suggested in the past for the alien gastropod *Cerithium scabridum* and the native *C. vulgatum*^54^, but this is yet to be demonstrated experimentally.

I suggest, by way of deduction and because all species collapses are not local but regional (i.e., they are evident along the entire 190 km-long Israeli coast, and some species probably beyond that; *Stramonita haemastoma* was not seen in Cyprus and Crete as well), and they occur over a range of taxonomic groups with very different biological and ecological traits, that the main driver(s) of many of these collapses is/are physical and operate on regional scales. From the varied sources of data and the longest regional *in-situ* offshore measurements^14^, I conclude that exceptional ocean warming, 4–10 times higher than the global average published in the recent IPCC report (0.11 °C per decade, ref. 55), has occurred along the Israeli coast. Accordingly, ocean warming, that is evidently intense in this region, is a prime suspect for many of those collapses. At least for one species, *P. lividus*, there is a strong experimental evidence that ocean warming on the Israeli coast was indeed detrimental. Recent lab and field experiments have demonstrated that this urchin dies when temperatures cross 30.5 °C, which occurs every summer on the Israeli coast since the late 1990 s^56^ (Figs 5 and 6), indicating that temperature is probably the main cause of its collapse. Competition for food with the invasive siganids likely also contributed by reducing the physiological state of the urchins making it more vulnerable to temperature impacts. Interestingly, high densities of *P. lividus* (up to 63 m^2^) were found in very recent urchin surveys (2013–15) near Alexandria (Egypt), to the southwest of the present study region^57^. In this area, the maximum summer temperatures were reported to be 29.5 °C^57^, still below the lethal temperature^56^. Unfortunately, for all but one of the collapsed native molluscs, *Ostera edulis*, I could not detect any published experiments on their physiological performance with relation to water temperatures, and *O. edulis* was only tested up to 25 °C^58^, which is not relevant to either past or present summer temperatures on the Israeli coast.

Evidence from the northwestern Mediterranean revealed several heat waves since the 1980 s that caused anomalous SST warming reaching the highest temperatures ever recorded in the region, between 1–3 °C above the climatic values. These heat waves caused mass mortality events of many sessile macro-invertebrate reef species^18^. In recent reviews and analyses, these and other events were related to climate change^59^,^60^. The reviews also revealed the lack of biological data that may demonstrate the impact of climate change in the Levant, a region where the most intense warming has occurred in the Mediterranean. Steady increase in SST, such as that occurring along the southeastern Mediterranean coast (although a notable upward shift in 1998 is evident), may result not only in temporary declines in abundance as in the case of a pulsed stress caused by heat waves, but rather by a gradual elimination of species from regions altogether, similar to what is already happening on land^61^,^62^. The species collapses documented in this study on the Levant coast may be a first evidence of that process (population collapses are an advanced stage of range contraction at the trailing edge of species distributional range ref. 63); in this case, some species seem to be at the verge of a regional extinction, and others may have completely disappeared.

Of course, it is likely that warming is only one component in an extensive multi-stressor environmental change in the eastern Mediterranean^64^ that is driving many of those collapses. The combination of stressors like warming and pollution, or warming and competition with alien species, may act additively or synergistically to considerably reduce the fitness and ultimately survival of sensitive species, especially in the already extreme Levant region, ending with severe population declines and regional extinctions. For example, *S. haemastoma* has been exposed to extensive TBT pollution (evidenced also by high imposex levels) all along the Israeli coast^65^ that may have hindered its reproduction (no recruitment was seen for five years during the late 1990 s^31^), and together with warming may have severely affected its population viability leading to its extirpation in the region. Competition with the Red Sea mussel, *B. pharaonis*, is probably an important cause of the major decline of the small Mediterranean mussel *Mytilaster minimus*, that was the dominant bivalve in the rocky intertidal until the 1990 s^66^, when the invader took over the habitat; but warming may have also contributed by favoring the invader (that was present in very low numbers in the southeastern Levant for 120 years until the 90 s^33^) while stressing the native. It is also likely that warming is not the direct cause for mortality in some cases but rather a trigger for the outbreak of disease^67^ that eventually eliminates a population, such as in the case of Caribbean corals^68^,^69^, or the California abalone^70^. For one of the large collapsed bivalve species, *Spondylus gaederopus*, there are two reports on mass mortalities in the western Mediterranean: one non-temperature related (and with no evidence of cause, but an epidemic was suggested) that occurred in the Columbretes Islands Marine Reserve (Spain) in summer 2005, and one that was temperature-related in Ischia (Italy) following a heat wave in 2009 (with maximum temperatures reaching 29 °C). The additive or synergistic role of climate change in a human-driven multi-stressor environment is an important new research direction that certainly deserves much attention by both ecologists and managers^71^,^72^.

The multi-species collapses reported in this study may be a sign for the initiation of a massive range-contraction of reef organisms that is unfolding, so far mostly unnoticed, in the Mediterranean Sea. Recent analysis of bottom-trawl data demonstrated the collapse of native species and domination of non-indigenous ones in soft-bottom Levant fish assemblages^73^. Although overfishing and competition with invasive species could be blamed for the considerable reduction in native fish abundances, the contribution of ocean warming in this ecosystem too cannot be ruled-out. New global climate velocity analysis (see Fig. 3 in ref. 74) shows that in the southeastern Levant, sea surface isotherms are “moving” fast to the northeast. This fast-warming area is therefore where one would expect to see sensitive native species (that live at the edge of their physiological comfort zone) gradually excluded by warming (and the urchin is one, but clear, evidence of that). Shifts in species distributional ranges are among the most profound expressions of climate change impacts on the earth’s biota^74^,^75^,^76^. So far, distributional shifts have been mostly established as species range expansions at the leading (cold) edge, in both terrestrial and marine systems^62^,^77^,^78^, while cases of contraction at the trailing edge has been understudied. The results present here may represent such a case.

Recent reviews of marine ecosystem and population declines^79^,^80^ supply little evidence that highly abundant coastal invertebrates have become consistently rare (>90% decline), perhaps with the exception of Caribbean coral reefs^81^. In the Mediterranean, historical trends of depletions and extirpations of economically and ecologically important species for which long-term records are available (the North Adriatic Sea) have not shown any recent invertebrate extinctions and only very few species have become rare^10^. However, regional models projected that the temperature increase should squeeze endemic Mediterranean, cold-water, (fish) species into smaller and smaller ‘cul-de-sac’ northwestern regions^82^. According to these bioclimatic envelope models, by the end of the century the contraction of suitable habitat will drive some of those endemic species towards extinction; but other models also surprisingly project an increase in (native fish) species richness in the Levantine Basin^83^, i.e., no meaningful species contraction in the area. Interestingly, in a recent global meta-analysis, benthic molluscs, on average, showed no range contraction at the trailing edge (see Fig. 2 in ref. 76), however, the analysis did not include the Levant region. Regardless of whether the population collapses shown here are a direct result of ocean warming or not, they are important at three levels: (1) this is the first evidence to show regional-scale (>150 km of coastline), multiple (tens of species), sustained (at least 5–6 years), collapses of abundant, non-harvested, invertebrate species from several trophic levels on non-tropical open coasts; (2) this is strong new evidence that the Levant is going through a considerable re-structuring (or even a phase shift) of its coastal communities, not only by massive species invasions but also by severe native species declines not related to harvesting; and (3) they could be a warning sign for similar population collapses that may soon occur further west in the Mediterranean and affect the structure, function and services of reef ecosystems. The collapse of *D. petraeum*, for example, means that the major ecosystem engineer of the vermetid reefs^84^ is currently ecologically-lost to the ecosystem, and that the integrity of the reefs themselves may thus be affected. One scenario, for instance, could be the extinction of the vermetid-reef ecosystem altogether because sea level that has already risen by 10.5 cm in the past two decades^85^ will continue to rise (predictions range up to 1 m by the end of the century) and inundate the current intertidal platforms, the present reefs will erode over time by the elements, and *D. petraeum* will not be there to create new ones at higher levels on the shore. As *D. petraeum* was suggested to regulate reef erosion and vermetid reefs may have an important regulating role of beach erosion (by protecting the sandy beach and coastal cliffs behind the reefs from wave action and storm surge), the extinction of the vermetid may mean, in the long run, the loss of this important ecosystem service. Clearly, there is an urgent need to follow those dramatic population collapses at the basin scale, clarify their drivers, test their ecosystem implications and learn from them about the possible future of global coastal biodiversity.

## Methods

### Focal species and their known distribution

Past quantitative data for any reef invertebrate species is extremely sparse for the southeastern Mediterranean shores. I have selected four species that are ecologically very important, are not harvested commercially in Israel and were known to be common, and for which at least one abundance data point from past decades exists. These included two gastropods and two urchins.

The large predatory gastropod (whelk) *Stramonita haemastoma* (Linnaeus, 1767) was considered a widely-spread species complex, but recent genetic analysis separated the complex to five different species^86^. The main distribution of the Mediterranean *S. haemastoma* includes, besides the whole Mediterranean, the Iberian coast, the west African coast down to Angola and the Macaronesian Islands (Azores and the Canaries), and a population also exists on the Venezuelan coast (Fig. 2d). Extracts from its hypobranchial gland (known as the “Tyrian purple”) were used for dyeing robes of Levantine royalty during biblical and Roman times (until 7th century AD, ref. 87). Hundreds of individuals were needed to supply enough dye for a single robe; therefore this whelk must have been extremely abundant historically. The species was known to occupy the rocky intertidal and shallow subtidal in wave-exposed areas, normally no deeper than 5 m^88^.

Two rocky reef sea urchins are considered common in the Mediterranean Sea, *Paracentrotus lividus* (Lamarck, 1816) and *Arbacia lixula* (Linnaeus, 1758). Both urchin species live on rocky reefs or seagrass, mostly in the shallow subtidal (down to 20–30 m) but can also be found in intertidal rockpools^89^. *P. lividus* is a widespread urchin^90^ found in the Mediterranean, the Irish Sea, the Iberian coast, and the west coast of Africa (Morocco), (Fig. 2e), known to be living in waters ranging from 10–29 °C^90^. It is considered a highly effective herbivore on Mediterranean benthic communities^91^. *A. lixula* (Linnaeus, 1758) has a similar distribution to *P. lividus*, but it does not extend to the Irish Sea, and has a population also in the Southern hemisphere on the coast of Brazil^92^.

*Dendropoma petraeum* (Monterosato, 1884) is a suspension-feeding, aggregative, vermetid gastropod, endemic to the Mediterranean, where it is found mostly in southern areas, forming intertidal horizontal reefs in only specific locations such as southeastern Spain, Malta, Sicily, a few sites in Crete and Cyprus, and most extensively in the southeastern Levant (Israel, Lebanon and Syria, Fig. 2f), in areas where the rock is soft and sedimentary^36^,^84^,^93^,^94^,^95^. Recent genetic analysis indicated that it, too, has four different clades that can be considered different species, one of which is a Levantine clade^96^,^97^. It forms a distinctive rim at the edge of vermetid reefs (abrasion platforms), shaping this unique habitat for other species^84^,^98^, thereby it can be considered an ecosystem engineer. *D. petraeum* is included in Appendix II (Strictly Protected Fauna Species) of the Bern Convention, and in Annex II (Endangered or Threatened Species) of the Protocol for Specially Protected Areas and Biodiversity in the Mediterranean (SPABIM Protocol, Barcelona Convention).

### Biological surveys

The present and past biological surveys described below have used different methodologies because the motivation behind them was different. Present day surveys are part of an extensive, long-term, ecological monitoring program that started in 2009 (intertidal) or several extensive surveys conducted between 2010–2015 (subtidal), both of which are mostly government-funded and are aimed to describe the macrobenthic community structure on the reefs (invertebrate, macroalgae and fish), and use general protocols designed to capture spatio-temporal changes in this community. Past surveys were part of student dissertations, or data collected sporadically but never published, and were much more restricted to a specific site or a specific organism, and the method used (e.g., quadrat or transect) depended on the observed density or distributional pattern of the studied organism.

### Present-day biological surveys

**Figure 1:**
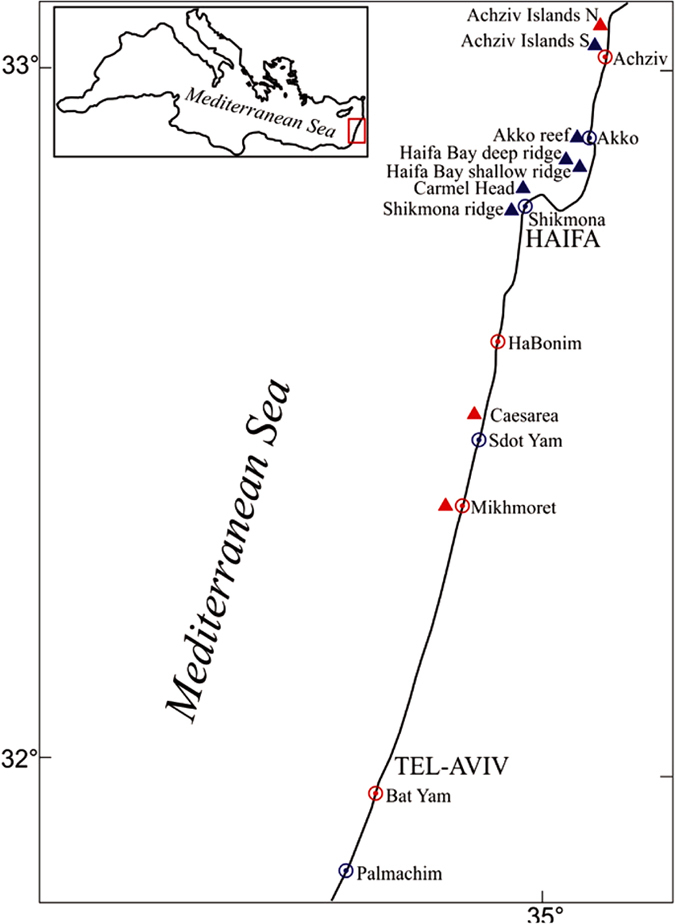
Map of main study areas (subtidal) and sites (intertidal) along the Israeli coast (triangles: offshore areas; circles: onshore sites. In red: sites with both past and present data). Created with CorelDraw cdr v5 (http://www.coreldraw.com).

**Figure 2:**
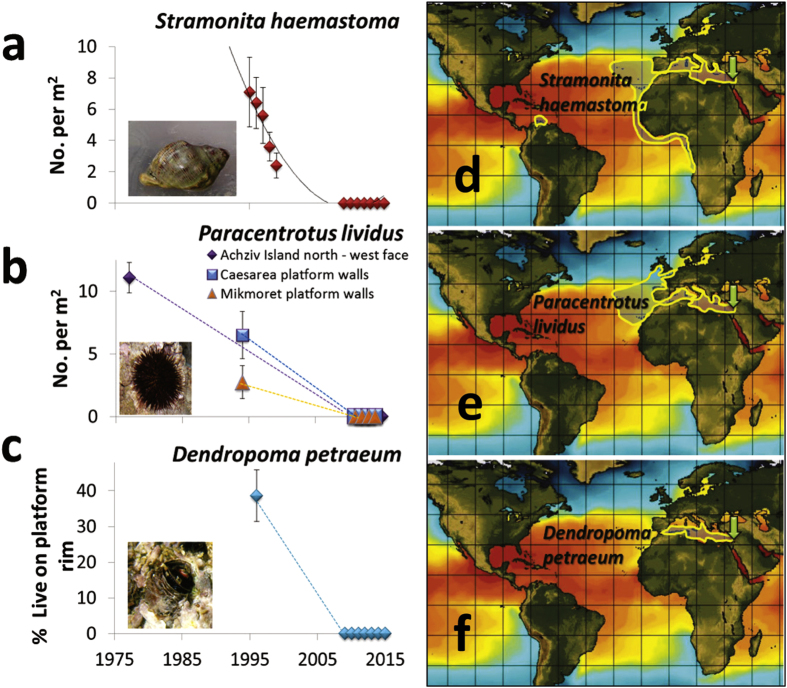
Changes in the abundance of three once highly-abundant rocky reef invertebrate species on the Israeli shore (**a–c**) and their distribution maps (**d–f**). Approximate distributional range of the three species (see Methods), shown as an area with yellow borders, overlain on a typical summer SST image downloaded from http://www.ssec.wisc.edu/data/sst/, provided courtesy of the Space Science and Engineering Center (SSEC), University of Wisconsin-Madison. Images derived using data from the NOAA National Centers for Environmental Prediction (NCEP). A green arrow indicates the southeastern Levant. (**a**) The density (average per m^2^ ± SE) of the whelk *Stramonita haemastoma* on intertidal rocks in Achziv, a site in the north of Israel where the highest densities were observed in the mid 1990 s; nearly eight individuals per m^2^ in 1995 were seen in this site, this number dropped by 3-fold until 1999 and not a single snail was found there between 2009–2015. The trend line is a polynomial fit. (**b**) The density (average per m^2^ ± SE) of the urchin *Paracentrotus lividus* on the west-facing walls of a small rocky island inside an enforced marine reserve (Achziv Islands N, density from 1976 is from old surveys done by Dothan^14^ at exactly the same location) and two sites in the center of the coast in the 1990 s and today. (**c**) The percent cover (average per meter of rim length ± SE) of the vermetid gastropod *Dendropoma petraeum* along the edge of an intertidal vermetid reef at Habonim on the central coast, where high live cover was still seen in the mid 1990 s. The dotted lines in b and c are only meant to demonstrate the general trend of reduction for the three species; the trajectory is unknown because of the large gap in data.

**Figure 3:**
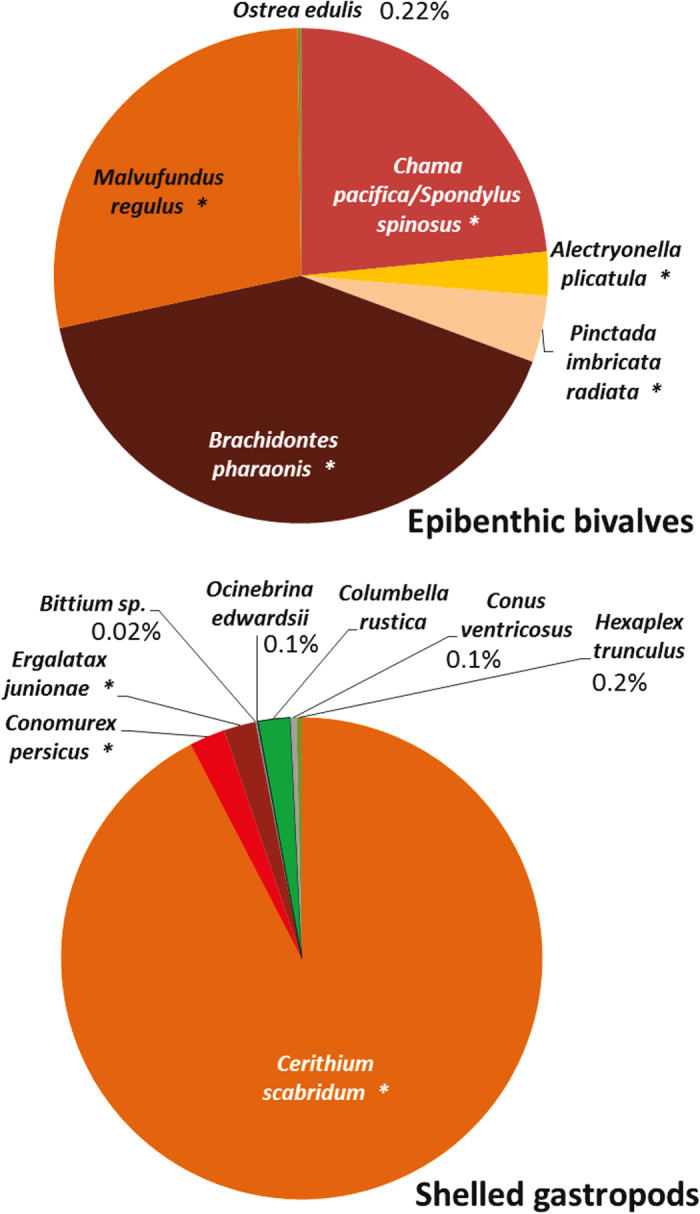
The summed relative abundance of non-indigenous (marked with an asterisk) and native bivalves and shelled gastropods. Counted during 2011–2014 on the north coast of Israel along 30 × 1 m transects during the visual surveys for macro-invertebrates on the subtidal reefs (2–25 m depth).

**Figure 4:**
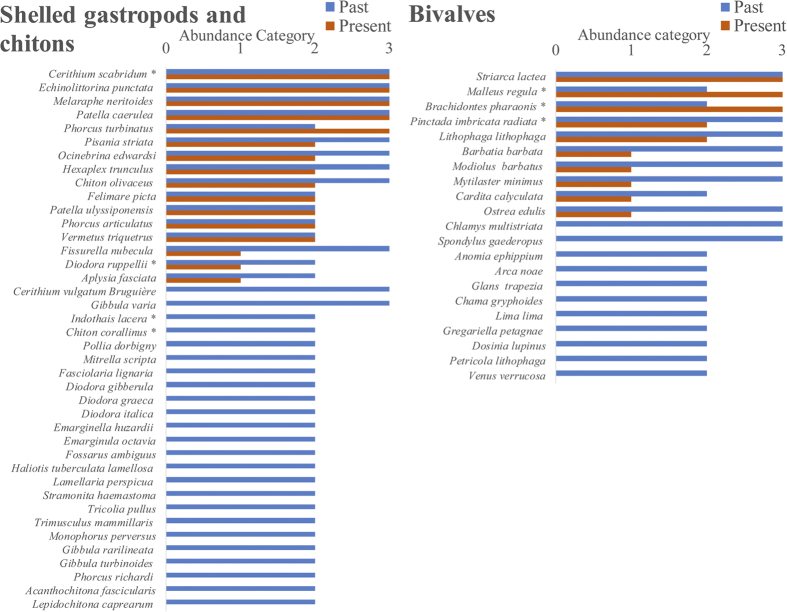
Past and present abundance categories of hard substrate (rocky reef) shelled gastropods and bivalves based on Levant taxonomic literature and present survey data. 0 = not found, 1 = rare, not common, 2 = common, 3 = very common. In present day surveys, rare or not common means that the species was seen in one or a few transects, or rarely in observations outside transects or in areas not surveyed, common means that the species was frequently seen but in low numbers, very common means that the species was frequently seen in high numbers. Species marked with asterisks are non-indigenous.

**Figure 5:**
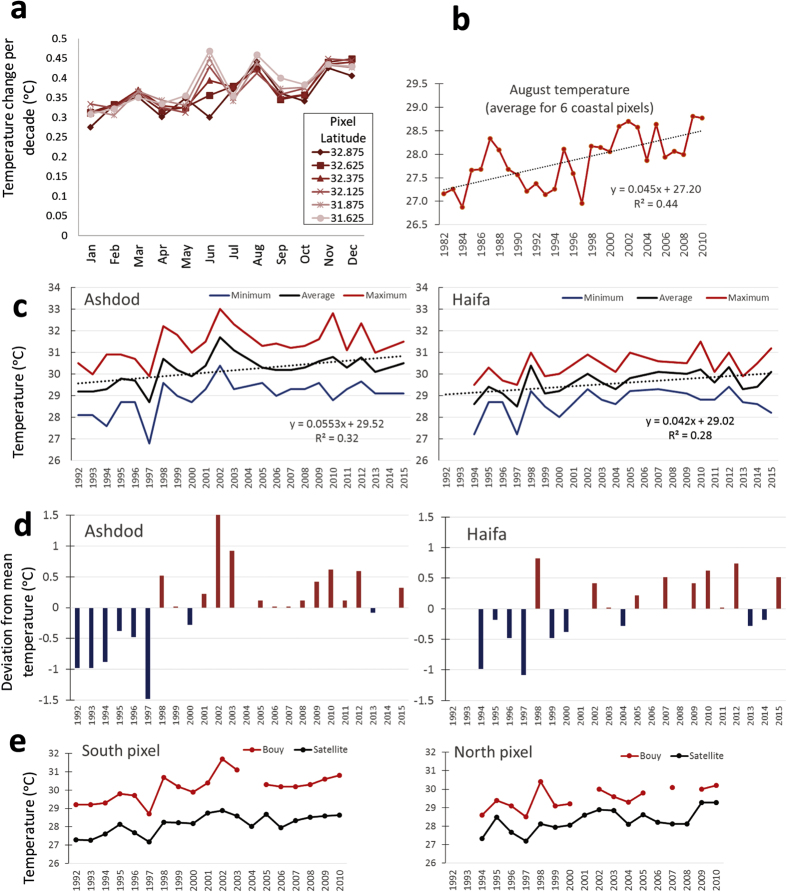
Evidence for sea surface temperature (SST) changes in the southeast Levant coastal waters. (**a**) Average SST-change per decade along the Israeli coast for every month of the year. Analysis was done from processed satellite data for six ¼ degree coastal pixels. Source is www.coastalwarming.com. (**b**) August temperatures averaged over the six coastal pixels. (**c**) Average, minimum and maximum August SST data from two buoys located 2 km offshore in Haifa (from 1994) in the north and Ashdod (from 1992) in the south. Data collected by the Israel Port Company and kindly provided by the Coastal and Marine Engineering Research Institute (CAMERI) of the Technion. (**d**) Deviation from the long-term mean of August temperatures for Ashdod and Haifa. (**e**) Comparison of the satellite and buoy data for a parallel measurement period for the south and north pixels where the buoys are located.

**Figure 6:**
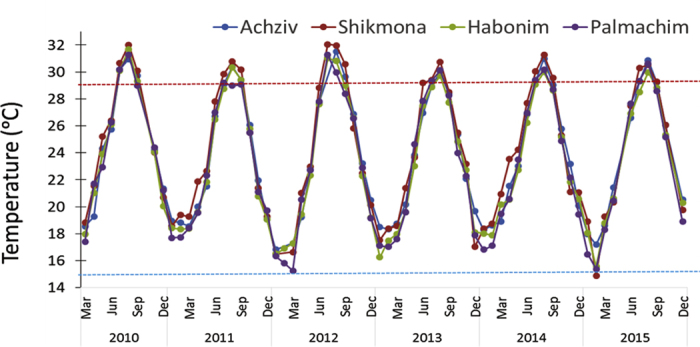
SST (at 0.5 m depth) interannual variability over a six-year period measured monthly at the four core intertidal monitoring sites from north to south: Achziv (AK), Shikmona (SK), Habonim (HB) and Palmachim (PL) (see site map in Fig. 1). Dotted horizontal lines represent typical peak summer and peak winter temperatures known for the coast 3–4 decades ago.

Between 2009–2015, the macrobenthic community (fish, invertebrates and macroalgae) at eleven intertidal sites spread along the Israeli coast, and 74 subtidal sites in several areas in the north (major sampling regions are shown in Fig. 1) was visually surveyed multiple times. Intertidally, the vermetid reefs in each site were sampled along four 50 m transect lines: at the platform seaward edge (the *D. petraeum* rim area), its center and its back (mid-midshore, high-midshore). The abundance of the macrobenthos (percent cover or count) was assessed in 15 random 50 × 50 cm quadrats along the transects. This was done annually during the fall since October 2009 at all 11 sites and seasonally at four core sites (382 transects in total). Subtidally, all large macro-invertebrates (including urchins and whelks) were counted along 30 × 1 m transects, and percent cover of different sessile space occupiers was assessed in fifteen 50 × 50 cm random photo-quadrats analyzed using the CPCe software^99^ in the lab. This was done between 2010–15 along 270 transects (at depths 1–25 m), in several main regions including: Haifa Bay, Carmel Head, Akko, the Achziv Islands, including the northern (now inside a marine reserve) and the southern (outside the reserve) islets that Dothan^32^ surveyed (see below). Qualitative surveys were also conducted at sites further south at shallow depths down to five meters to search for urchins and *S. haemastoma*. Two other regions in the eastern basin and one in the western basin were also surveyed for comparison using identical methodologies. Intertidal and subtidal surveys were conducted in June 2013 in Crete (four sites) and Cyprus (four sites), and subtidal surveys (seven sites) were conducted in September 2014 near Naples and around the nearby island of Ischia in southern Italy.

When conducting the visual surveys in the field or the photoquadrat analysis in the lab there is a great deal of “hidden” diversity that is missed in areas of high structural complexity, mostly that of cryptic and encrusting animals, and macrofauna that live inside burrows and crevices in the rocks. To fill that gap in sampling (mostly for mollusca), in autumn 2011 and spring 2012, fifty randomly-selected “live rocks” (rocks that are either porous or biogenic and are covered and burrowed by macrobenthic organisms), 1–2 kg in weight, were broken-off the substrate at 5–15 m depth in the Carmel Head and Haifa Bay area for a full macrobenthic assessment. These rocks were brought live to the lab where they were carefully fragmented with a chisel and hammer and the animals removed and counted.

### Past biological survey data

Dothan^32^, in 1976, conducted macrobenthic surveys (including urchins) around two small rocky islets (known as Achziv Islands) off the northern Achziv coast near the Lebanese border (Fig. 1) down to 25 m, using 1 × 1 m quadrats, overall sampling 126 quadrats. In 1994, I surveyed several shallow (<5 m) rocky sites along the central coast, including Mikhmoret and Caesarea (near Sdot Yam, Fig. 1) for mobile macrobenthic organisms inside 1 × 1 quadrats (on walls, where density appeared relatively high) or along 10 × 1 m transects (on horizontal surfaces, where density appeared very low). Sampling *S. haemastoma* populations was conducted between 1995–99 at 11 sites along the entire Israeli coast (Fig. 1) using different methods depending on density^31^. In 1996, *D. petraeum* was quantitatively sampled only at one site on the central coast, Habonim. A transect line was placed along the seaward edge of the platform where the rim was located, and the percentage of live *Dendropoma* cover of each meter along 50 m of the edge was measured and then averaged. In other sites, the state of the rim of *D. petraeum* was only qualitatively assessed^33^.

### Malacological taxonomic literature and archived museum records

For comparison of present-day survey data on molluscan abundance to past information on this group, relevant species were searched for in the very detailed annotated list of Israeli Mediterranean coast molluscs^29^,^30^. The list is based on literature summary and extensive field surveys conducted during 1967–1972 and 1974–1977 along the Israel continental shelf. The text also mentions the geographic distribution of all species and their categories of abundance (rare, uncommon, common, very common), based on Fretter & Graham^100^. In order to avoid possible detection bias when doing the past-present comparison, a short-list was made of the species that are (1) large enough to be easily seen in our surveys (>1 cm), (2) were specified in the publication as living on hard substrate in the intertidal or shallow subtidal, (3) are mentioned to have been recorded live (with the soft tissue) and (4) are described as common or very common. Only this list of species was compared to the present day survey abundance data because species that were rare then can be still be missed in today’s surveys.

Information from Museum taxonomic collections was used to confirm if any of the species that were not found in the present-day surveys were nonetheless reported alive on the coast since 2010. H. Mienis, the curator of the National Mollusc Collections at the Hebrew University and the Steinhardt Museum of Natural History and National Research Center in Tel Aviv University, was given the short-list mentioned above, and for each species, the year when a live specimen of the species was last brought to the collections was reported.

### Local knowledge survey

I used a local-knowledge survey only for urchins. This is because the other invertebrate taxa in this study are most probably much less noticeable by layman if not specifically looked for, and are less familiar by the general public, and thus information on them would be much less reliable. A simple, four question multiple-answer survey was designed to characterize possible trends in urchin populations, their timing and the characterization of the responders (experience and background, see Supplementary Methods S1). Considering the imperfections in relying on memory, rather than providing specific dates and sightings, responders were asked to refer to long time-periods (decades). The survey was circulated in August 2015, using the internet to publish it among groups who should be familiar with the local marine life (i.e., amateur and professional fishermen and divers as well as marine scientists). Analysis is based only on responders who declared 10 years or more of familiarity with the Mediterranean coast underwater world.

### Temperature trends

#### Satellite data

The Coastal Warming project^40^ used the NOAA Optimum Interpolation ¼ Degree AVHRR Daily Sea Surface Temperature (SST) analysis from 01-01-1982 to 12-31-2010 to map worldwide changes in coastal SST (the line of the 25 × 25 km pixels most adjacent to the shoreline) over the last three decades (see http://coastalwarming.com). I used a subset of the products of this data to look for patterns in SST change along the eastern Mediterranean Israeli coast over that period (six ¼ degree pixels cover the ~190 km Israeli coast) for each month of the year.

#### *In-situ* nearshore temperature data

Monthly average, minimum and maximum values of nearshore SST measured by loggers on buoys (operated by Israel Port Company) located 2 km offshore in Ashdod (south coast, close to the Palmachim core site) and Haifa (north coast, close to the Shikmona core site) for the periods 1992–2015 and 1994–2015, respectively, were obtained from the Coastal and Marine Engineering Research Institute (CAMERI) of the Technion Institute of Technology. The remote sensing temperature data (courtesy of Fernando Lima who provided the raw data) of the north and south coastal pixels was compared to the buoy data for the same period (1992–2010) to examine how similar and correlative they are. Very-nearshore SST were measured monthly at 0.5 m depth with a multi-probe (YSI 556MPS) at four core monitoring sites: Achziv, Shikmona, Habonim and Palmachim (see study sites in Fig. 1). Temperature loggers (HOBO TidBits V2) were deployed on the rocks at 0.5 m depth at the same sites and measured temperatures hourly over a five-year period. These measurements were temporally fragmented (and are therefore not shown) but the available data was highly consistent with the monthly YSI sampling.

## Additional Information

**How to cite this article**: Rilov, G. Multi-species collapses at the warm edge of a warming sea. *Sci. Rep.* **6**, 36897; doi: 10.1038/srep36897 (2016).

**Publisher’s note**: Springer Nature remains neutral with regard to jurisdictional claims in published maps and institutional affiliations.

## Supplementary Material

Supplementary Information
