# Supplementary material for: Multi-species collapses at the warm edge of a warming sea
Source: Sci Rep. 2016 Nov 17;6:36897. doi: 10.1038/srep36897 (PMC5113072; doi:10.1038/srep36897)
Supplement: Supplementary Information [file srep36897-s1.pdf]

Multi-species collapses at the warm edge of a warming sea

Gil Rilov

Table S1 List of mollusca species annotated as common or vary common in Barash and Danin (1992) publication and their present day status.

Class	Family	Species	Vertical distribution zone From Barash and Danin 1982	Global distribution from Darash and Danin 1982	Native\ Non-native	Described abundance (Barash and Danin 1982)	Currant abundance (Rilov Lab data)	latest year of live record
Bivalvia	Anomiidae	*Anomia ephippium*	Intertidal zone, Infralittoral zone	Mediterranean (east, west), East Atlantic (Iceland, Norway, British Is., France, Spain, Portugal, Morocco, Senegal, guinea, Gabon, south Africa, Madeira, Cape Verde Is. , sea of Marmara.	Native	common	not found	Ashdod, 30.01.2012
Bivalvia	Arcidae	*Arca noae*	Intertidal, subtidal (3-91 m)	Mediterranean (east, west), Sea of Marmara, East Atlantic (Spain-Angola-Canaries)	Native	common	not found	<1999
Bivalvia	Arcidae	*Barbatia barbata*	Intertidal, subtidal (2-27 m)	Mediterranean (east, west), Sea of Marmara, East Atlantic (Spain to -Morocco, Cape Verde Island)	Native	very common	rare (on rocks)	<1999
Bivalvia	Carditidae	*Cardita calyculata*	Intertidal, subtidal (3-91 m)	Mediterranean (east, west), East Atlantic (Spain-Morocco-Canaries)	Native	common	rare	Hadera Power Plant, 2005
Bivalvia	Carditidae	*Glans trapezia*	Intertidal, subtidal (18-79 m)	Mediterranean (east, west), East Atlantic (Spain-Portugal)	Native	common	not found	Segavyon I., 18.10.2004

Bivalvia	Chamidae	*Chama gryphoides*	Intertidal, subtidal (4-200 m)	Mediterranean (east, west), East Atlantic (French-Morocco-Canaries)	Native	common	not found	Ashdod, 30.01.2012
Bivalvia	Limidae	*Lima (Lima) lima*	Intertidal, subtidal (2-63 m)	Mediterranean (east, west), East Atlantic (Spain to Morocco, Canaries), West Atlantic (Florida- West Indies), Indian Ocean (Gulf of Suez, Red Sea, East Africa) Pacific Ocean (Japan, New Caledonia)	Native	common	not found	<1999
Bivalvia	Malleidae	*Malvufundus regulus **	Intertidal, subtidal (2-96 m)	Mediterranean (east), Lebanon, Syria, Cyprus, South Turkey, Hawaii, Suez channel	Non-native	common	very common	Sedot Yam, 06.2012
Bivalvia	Mytilidae	*Gregariella petagnae*	Intertidal zone, infralittoral zone	Mediterranean (east, west), East Atlantic (France-Spain-Portugal, Morocco, Senegal, Guinea), Alboran sea	Native	Moderately common	not found	N/A
Bivalvia	Mytilidae	*Lithophaga lithophaga*	Intertidal zone, Infralittoral zone	Mediterranean (east, west), East Atlantic (France-Spain-Portugal, Morocco, Senegal,), Indo pacific : Aden, Persian Gulf	Native	very common	common	Akko, 24.06.2012
Bivalvia	Mytilidae	*Modiolus barbatus*	Intertidal zone, Infralittoral zone	Mediterranean (east, west), East Atlantic (France-Spain-Portugal, Morocco, Senegal, Cape Verde Is.), Black sea, sea of Marmara.	Native	very common	not common	Tel Baruch, 25.01.2012

Bivalvia	Mytilidae	*Mytilaster minimus*	Intertidal zone, infralittoral fringe	Mediterranean (east, west), East Atlantic (France-Spain-Portugal, Morocco), Alboran sea	Native	very common	not common	Tel Baruch, 20.12.2012;
Bivalvia	Mytilidae	*Brachidontes pharaonis* *	Intertidal	Mediterranean (east, west), Indo-Pacific (Suez canal and bays, Aqaba, Red Sea, Gulf of Aden, Somalia, Southern Africa, Natal, Cape of good Hope, Madagascar, Australia)	Non-native	common	very common	Everywhere
Bivalvia	Noetiidae	*Striarca lactea*	Intertidal, subtidal (5-192 m)	Mediterranean (east, west), Black Sea, East Atlantic (British Isle-Cape of good Hope), Indian Ocean (India, Red Sea)	Native	very common	very common	Commonly encountered everywhere
Bivalvia	Ostreidae	*Ostrea edulis*	Intertidal, subtidal (2-31 m)	Mediterranean (east, west), Black Sea, Sea of Marmara, East Atlantic (Norway-Morocco)	Native	very common	not found	<1999
Bivalvia	Pectinidae	*Chlamys multistriata*	Intertidal, subtidal (113-180 m)	Mediterranean (east, west), Sea of Marmara, East Atlantic (Norway-Senegal)	Native	very common	not found	<1999
Bivalvia	Pteriidae	*Pinctada imbricata radiata* *	Intertidal, subtidal (2-69 m)	Mediterranean (east, west), IndoPacific (Red Sea, Gulf of Eilat and Suez, Persian Gulf, Somalia, Madagascar, South Africa, New Guinea, Hawaii Islands, Australia, New Zealand	Non-native	very common	common	Nahsholim, 20.12.2012
Bivalvia	Spondylidae	*Spondylus gaederopus*	Subtidal (7-20 m)	Mediterranean (east, west), East Atlantic (Portugal - Gianna, Canaries)	Native	very common	not found	<1999

Bivalvia	Veneridae	*Dosinia lupinus*	Subtidal (5-85 m)	Mediterranean (east, west), Sea of Marmara, East Atlantic (Iceland- Ivory Coast, Canaries)	Native	common	not found	N/A
Bivalvia	Veneridae	*Petricola lithophaga*	Intertidal, Infralittoral zone (to 6 m)	Mediterranean (east, west), East Atlantic (British Is., France, Spain, Portugal, Morocco) black sea , sea of Marmara	Native	common	not found	Haifa Power Station Inlet, 27.06.2005
Gastropoda	Aplysiidae	*Aplysia fasciata*	Intertidal, subtidal (9-24 m)	Mediterranean (east, west), East Atlantic (England-Angola), IndoPacific (Gulf of Aqaba-Red Sea)	Native	common	not common	N/A
Gastropoda	Buccinidae	*Pisania striata*	Intertidal	Mediterranean (east, west), Sea of Marmara, East Atlantic (Spain-Morocco)	Native	very common	common	Tel Baruch, 17.08.2012
Gastropoda	Buccinidae	*Pollia dorbigny*	Intertidal, subtidal (7-37 m)	Mediterranean (east, west), East Atlantic (Spain-Republic of Ghana)	Native	common	not found	Shikmona, 09.05.2003
Gastropoda	Cerithiidae	*Bittium reticulatum*	Subtidal (not mentioned on rocks)	Mediterranean (east, west), East Atlantic (Norway to Morocco and the islands)		very common	very common	N/A
Gastropoda	Cerithiidae	*Cerithium scabridum* *	Intertidal	Mediterranean (east, west), IndoPacific (Red Sea, Gulf of Eilat and Suez -Persian Gulf- South India)	Non-native	very common	very common	Tel Baruch, 17.08.2012
Gastropoda	Cerithiidae	*Cerithium vulgatum Bruguière*	Intertidal, subtidal (to 2 m)	Mediterranean (east, west), East Atlantic (Spain-Morocco, Canaries)	Native	very common	not found	<1999

Gastropoda	Chromodorididae	*Felimare picta*	Intertidal, subtidal (2-46 m)	Mediterranean (east, west), East Atlantic (Spain, French, Canaries-Gianna)	Native	common	common	N/A
Gastropoda	Cloumbellidae	*Mitrella scripta*	Intertidal, subtidal (46-91 m)	Mediterranean (east, west), Sea of Marmara, East Atlantic (Spain-Morocco-Madeira)	Native	common	not found	N/A
Gastropoda	Fasciolariidae	*Fasciolaria lignaria*	Intertidal, subtidal (to 3m)	Mediterranean (endemic)	Native	common	not found	<1999
Gastropoda	Fissurellidae	*Diodora gibberula*	Intertidal	Mediterranean (east, west), Black Sea, East Atlantic (Spain-Cape Verde)	Native	common	not found	Bat Yam, 21.02.2002
Gastropoda	Fissurellidae	*Diodora graeca*	Intertidal, subtidal (to 75 m)	Mediterranean (east, west), Black Sea, East Atlantic (England - Canaries)	Native	common	not found	<1999
Gastropoda	Fissurellidae	*Diodora italica*	Intertidal	Mediterranean (endemic)	Native	common	not found	Tel Baruch, 25.03.2013
Gastropoda	Fissurellidae	*Diodora ruppellii* *	Intertidal, subtidal (to 70 m)	Mediterranean, IndoPacific	Non-native	common	not common	Apolonia, 21.02.2012
Gastropoda	Fissurellidae	*Emarginella huzardii*	subtidal (18-42 m)	Mediterranean (east, west), East Atlantic (Morocco, Canaries)	Native	common	not found	<1999
Gastropoda	Fissurellidae	*Emarginula octavia*	Intertidal, subtidal (22-55 m)	Mediterranean (east, west), East Atlantic (Morocco, Canaries), IndoPacific (India)	Native	common	not found	<1999
Gastropoda	Fissurellidae	*Fissurella nubecula*	Intertidal	Mediterranean (east, west), East Atlantic	Native	very common	not common	Apollonia, 31.03.2010
Gastropoda	Fossaridae	*Fossarus ambiguus*	Intertidal	Mediterranean (east, west), East Atlantic (French-Angola)	Native	common	not found	N/A

Gastropoda	Haliotidae	*Haliotis tuberculata lamellosa*	Intertidal, subtidal (to 23 m)	Mediterranean (east, west), East Atlantic (Spain to Morocco, Canaries)	Native	common	not found	<1999
Gastropoda	Lamellariidae	*Lamellaria perspicua*	Intertidal,	Mediterranean (east, west), East Atlantic (Iceland, Norway- Senegal, South Africa), West Atlantic (Florida, Caribbean, Brazil, Patagonia), East Pacific (Mexico, Chile)	Native	common	not found	<1999
Gastropoda	Littorinidae	*Echinolittorina punctata*	Intertidal	Mediterranean (east, west), East Atlantic (Spain-South Africa)	Native	very common	very common	Tel Baruch, 17.08.2012
Gastropoda	Littorinidae	*Melaraphe neritoides*	Intertidal	Mediterranean (east, west), Black Sea, Sea of Marmara, East Atlantic (England-Morocco-Canaries)	Native	very common	very common	Tel Baruch, 17.08.2012
Gastropoda	Muricidae	*Indothais lacera* *	Intertidal, subtidal (to 26 m)	Mediterranean (east, west), Persian Gulf, Sri Lanka, India, Siam, Indonesia)	Non-native	common	not found	Rishon leZiyyon, 21.12.2003
Gastropoda	Muricidae	*Ocinebrina edwardsi*	Intertidal, subtidal	Mediterranean (east, west), Sea of Marmara, East Atlantic (French-Canaries)	Native	very common	common	N/A
Gastropoda	Muricidae	*Stramonita haemastoma*	Intertidal, subtidal (to 9 m)	Mediterranean (east, west), East Atlantic (Spain to Cape Verde Island), West Atlantic (North America and Caribbean- Brazil)	Native	common	not found	<1999
Gastropoda	Muricidae	*Hexaplex trunculus*	Intertidal, subtidal	Mediterranean (east, west), East Atlantic (Spain-Canaries)	Native	very common	common	<1999

Gastropoda	Patellidae	*Patella ulyssiponensis*	Intertidal	Mediterranean (east, west), Black Sea, Atlantic (Norway-Canaries)	Native	common	common	Akhziv, 16.03.2011
Gastropoda	Patellidae	*Patella caerulea*	Intertidal	Mediterranean (east, west), East Atlantic (Portugal - Canaries)	Native	very common	very common	Nahsholim, 25.03.2013
Gastropoda	Phasianellidae	*Tricolia pullus*	Intertidal, subtidal (to 4 m)	Mediterranean (east, west), Black Sea, Sea of Marmara, East Atlantic (England - Senegal-Canaries)	Native	common	not found	N/A
Gastropoda	Trimusculidae	*Trimusculus mammillaris*	Intertidal	Mediterranean (east, west), East Atlantic (Portugal-Congo, Cape Verde Island)	Native	common	not found	Shikmona, 15.09.2001
Gastropoda	Triphoridae	*Monophorus perversus*	Intertidal, subtidal (18-191 m)	Mediterranean (east, west), Black Sea, Sea of Marmara, East Atlantic (Norway-Senegal and Canary Islands)	Native	common	not found	N/A
Gastropoda	Trochidae	*Gibbula rarilineata*	Intertidal	Mediterranean (endemic)	Native	common	not found	<1999
Gastropoda	Trochidae	*Gibbula turbinoides*	Intertidal, subtidal (to 22 m)	Mediterranean (endemic)	Native	common	not found	Shikmona, 09.05.2003
Gastropoda	Trochidae	*Gibbula varia*	Intertidal, subtidal (to 4 m)	Mediterranean (east, west), Black Sea, East Atlantic (Spain-Portugal)	Native	very common	not found	Ashdod, 01.07.2010
Gastropoda	Trochidae	*Gibbula ardens*	Intertidal, subtidal (to 22 m)	Mediterranean (east, west), East Atlantic	Native	very common	not found	
Gastropoda	Trochidae	*Gibbula umbilicaris*	Intertidal			common	not found	N/A
Gastropoda	Trochidae	*Phorcus articulatus*	Intertidal	Mediterranean (east, west), Sea of Marmara, East Atlantic (Spain to - Morocco, Cape Verde Island)	Native	common	common	Shikmona, 2013

Gastropoda	Trochidae	*Phorcus richardi*	Intertidal	Mediterranean (east, west), East Atlantic (Portugal-Canaries)	Native	common	not found	<1999
Gastropoda	Trochidae	*Phorcus turbinatus*	Intertidal	Mediterranean (east, west), East Atlantic (Spain--Morocco, Canaries)	Native	common	very common	Tel Baruch, 18.03.2005
Gastropoda	Vermetidae	*Vermetus triquetrus*	Intertidal	Mediterranean (east, west), East Atlantic (Spain-Morocco, Canaries)	Native	common	common	Shahaf I., 08.05.2007
Gastropoda	Vermetidae	*Thylacodes arenarius*	Intertidal, subtidal (18-70m)	Mediterranean (east, west), East Atlantic (Portugal, Morocco, Madeira)	Native	very common	not found	N/A
Gastropoda	Vermetidae	*Dendropoma petraeum*	Intertidal, subtidal (1 m)		Native	common	rare	N/A
Polyplacophora	Acanthochitonidae	*Acanthochitona fascicularis*	Intertidal, deep subtidal	Mediterranean (east, west), East Atlantic (from the English Channel, Africa, Canaries)	Native	common	not found	<1999
Polyplacophora	Chitonidae	*Chiton corallinus* *	Deep subtidal	Mediterranean (east, west), East Atlantic (Morocco), IndoPacific (Red Sea)	Non-native	common	not found	<1999
Polyplacophora	Chitonidae	*Chiton olivaceus*	Intertidal, subtidal (to 26 m)	Mediterranean (east, west), East Atlantic (Morocco, Portugal, Spain)	Native	very common	common	<1999
Polyplacophora	Lepidochitonidae	*Lepidochitona caprearum*	Intertidal	Mediterranean (east, west), Black Sea, Atlantic (Morocco, Portugal)	Native	common	not found	<1999
